# Synthetic Evaluation of MicroRNA-1-3p Expression in Head and Neck Squamous Cell Carcinoma Based on Microarray Chips and MicroRNA Sequencing

**DOI:** 10.1155/2021/6529255

**Published:** 2021-08-24

**Authors:** Yubing Chen, Mingjiang Liu, Hu Jin, Bo Peng, Luo Dai, Sifan Wang, Hao Xing, Baoju Wang, Zhan Wu

**Affiliations:** ^1^Division of Hepatobiliary Surgery, The First Affiliated Hospital of Guangxi Medical University, Nanning, Guangxi, China; ^2^Department of Pathology, Xiangyang Central Hospital, Xiangcheng District, Xiangyang, Hubei, China

## Abstract

**Background:**

MicroRNA-1-3p (miR-1-3p) exerts significant regulation in various tumor cells, but its molecular mechanisms in head and neck squamous cell carcinoma (HNSCC) are still ill defined. This study is aimed at detecting the expression of miR-1-3p in HNSCC and at determining its significant regulatory pathways.

**Methods:**

Data were obtained from the Cancer Genome Atlas (TCGA), Gene Expression Omnibus (GEO), Oncomine, ArrayExpress, Sequence Read Archive (SRA) databases, and additional literature. Expression values of miR-1-3p in HNSCC were analyzed comprehensively. The R language software was employed to screen differentially expressed genes, and bioinformatics assessment was performed. One sequence dataset (HNSCC: *n* = 484; noncancer: *n* = 44) and 18 chip datasets (HNSCC: *n* = 656; noncancer: *n* = 199) were obtained.

**Results:**

The expression of miR-1-3p in HNSCC was visibly decreased in compare with noncancerous tissues. There were distinct differences in tumor state (*P* = 0.0417), pathological stage (*P* = 0.0058), and T stage (*P* = 0.0044). Comprehensive analysis of sequence and chip data also indicated that miR-1-3p was lowly expressed in HNSCC. The diagnostic performance of miR-1-3p in HNSCC is reflected in the sensitivity and specificity of the collection, etc. Bioinformatics analysis showed the possible biological process, cellular component, molecular function, and KEGG pathways of miR-1-3p in HNSCC. And *ITGB4* was a possible target of miR-1-3p.

**Conclusions:**

miR-1-3p's low expression may facilitate tumorigenesis and evolution in HNSCC through signaling pathways. *ITGB4* may be a key gene in targeting pathways but still needs verification through in vitro experiments.

## 1. Introduction

HNSCC is a human malignant neoplasm common in certain regions. The morbidity of HNSCC has improved dramatically in recent years, particularly among women. Each year, over 600,000 patients are diagnosed HNSCC globally, over half in the Asia-pacific regions [1]. Platinum-based chemotherapy, radiotherapy, and cetuximab are commonly used to treat recurrent/metastatic HNSCC in Asia [2]. Unfortunately, about 75% of these patients have local progression (60%) or metastasis (15%) upon first contact, and the median survival of patients with relapse and/or metastasis is only 6 months. Survival rates drop rapidly in patients who fail first-line treatment, often dying within three months [3]. Seeking antitumor therapies that are more effective, more scholars are paying close attention to molecular-targeted therapy. Recently, many studies on microRNAs (miRNAs) have been affirmed in the cancer field, and molecular-targeted therapy has become a burgeoning treatment for tumors [4].

MicroRNAs of approximately 22 nucleotides long are noncoding single-stranded RNAs, coded efficiently by endogenous genes [5]. They regulate the expression of posttranscription genes. Many studies have presented distinctions in the expression of some miRNAs in cancerous and noncancerous tissues [6], utilizing signaling pathways to control key genes affecting physiological and biochemical processes such as proliferation and differentiation in tumor cells. Notwithstanding, the clinical value and molecular functions of individual miRNAs remain relatively unexplored. As a gene member, miRNA-1-3p targets different proteins or genes and affects the occurrence and development of gastric [7], colon [8], and breast carcinomas [9, 10], among others [11–13]. However, there are few relevant articles about the expression of miR-1-3p in HNSCC. Only 8 research teams have explored the ontology of miR-1-3p on key genes that regulate HNSCC [1, 14–20]. Potential molecular mechanism regulation in HNSCC is still unclear, and new enrichment regulatory pathways need to be proposed.

**Figure 1:**
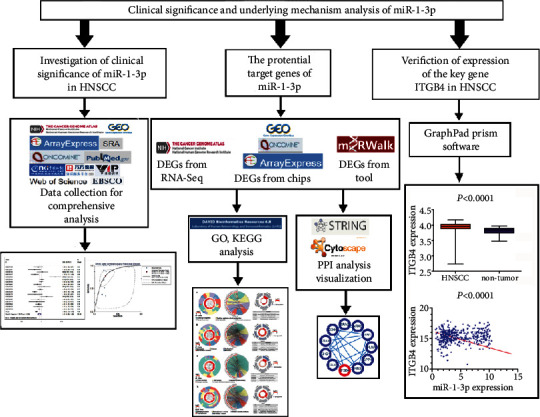
The research technical circuit diagram of this study.

In this study, a total of 1,140 cancer samples and 243 noncancer samples were collected based on sequencing, microarray, and literature data to probe the clinical significance and impact of miR-1-3p in HNSCC. Potential molecular mechanisms, including significant genes and enrichment paths of miR-1-3p in HNSCC, were summarized. *ITGB4* was one of the target genes. The schematic of the research design is shown in Figure 1.

## 2. Materials and Methods

### 2.1. Sources of miR-1-3p Expression Data in HNSCC

#### 2.1.1. MicroRNA-Seq Data

The miRNA sequence dataset was obtained from TCGA [21], including samples of cancerous and noncancerous tissues. The steps for data download were as follows: the site for UCSC-Xena (https://xena.ucsc.edu/) visited, checked “TCGA hub” in the “DATASETS” option, and selected “TCGA(HNSC),” which contains 25 datasets. In the new interface, selected “IlluminaHiseq (*n* = 529) TCGA hub” to download matrix files and gene annotation files. By matching the two, the expression value of miR-1-3p was determined. After deleting missing data, mature miR-1-3p expression data were extracted. Meanwhile, using “sangerbox,” the corresponding clinical case data were downloaded for analysis.

#### 2.1.2. Microarray Data

Microarrays were filtered to evaluate miR-1-3p expression in Gene Expression Omnibus (GEO) [22]. The overall strategy for retrieval was OSCC OR HNSCC OR “head and neck” OR “nasopharynx.” We adjusted search terms to achieve the best range. The search was restricted to “Series” in “Entry type” and “H. sapiens” in “organisms.” All research contained in the chips followed these criteria: (1) the species is human, (2) the objective is tissue, (3) microarray results include required gene, (4) containing both HNSCC tissues and nontumor tissues, and (5) miR-1-3p expression is mature. Conversely, the exclusion criteria were (1) objects other than human, (2) serum sample or other liquid types, (3) the chip does not contain the required genes, (4) there is only the experimental group or the control group, (5) control samples have other related diseases, and (6) include drug-related research or other unrelated interventions. After filtering the chips, chip number was input into the GEO database for querying detailed data; we downloaded the “SOFT formatted family file(s)” probe annotation files and “Series Matrix file(s)” gene probe expression Matrix file, to find the miR-1-3p corresponding expression data. For chip screening in ArrayExpress [23], Oncomine [24], and SRA [25], the methods were the same as above.

#### 2.1.3. Literature Data

We reviewed articles about gene expression in HNSCC in Chinese and foreign scientific research sites, including CNKI, Wanfang, Vip, PubMed, Web of Science, and EBSCO databases. We extracted these chips to supplement existing microarrays to acquire unabridged data.

### 2.2. Comprehensive and Detailed Analysis

Based on the sequencing data and the specific content of each microarray, the data were selected to make log2 processing or not. The mean expression level (mean) and standard deviation (SD) were calculated using SPSS 22.0. The “car” package of R was employed to draw violin plots to clarify that the miR-1-3p's expression was different in cancerous and noncancerous tissues. To evaluate the expression level of miR-1-3p comprehensively, a meta-analysis of continuous variables was conducted with Stata 12.0. When heterogeneity was small (*I*^2^ < 50%), the fixed effect model was adopted for analysis. On the contrary, if heterogeneity was extensive (*I*^2^ > 50%), a random-effect model was used, and we continued to perform sensitivity analysis to determine the sources of heterogeneity. After removing the chips that contributed to heterogeneity, we reanalyzed the results based on remaining data. If heterogeneity was less than 50%, the results were reliable. For statistical analysis, if the standard mean deviation (SMD) < 0 and the 95% confidence interval (CI) did not cross the 0-point coordinate line, the gene was demonstrated to be significantly lower in HNSCC. An SMD > 0 meant that the research object was highly expressed in carcinoma. The ROC curves were plotted using SPSS, and the sROC curve was drawn using the Stata software to forecast the clinical significance of miR-1-3p in HNSCC. When the area under the curve was >0.5, this index had a certain diagnostic value for diseases. The AUC over 0.7 indicated a good diagnostic value. Publication bias was determined using Begg's funnel plot.

### 2.3. Screening of Differential Genes

Based on log  | FC | >1 and *P* < 0.05, 39 arrays of the expression of long noncoding genes in HNSCC and 3 miR-1-3p transfection samples (GSM610393, GSM610394, GSM639297) were selected from TCGA, GEO, Oncomine, and ArrayExpress databases. DEGs that appeared over 8 times were singled out from 39 datasets, combined with miR-1-3p transfected samples and the prediction tool (miRWalk 2.0) to form final differential genes of HNSCC.

### 2.4. Bioinformatics Analytical Methodology

Gene annotation and pathway enrichment analysis of DEGs were conducted in DAVID v 6.8; top-ranked annotations and pathways were listed, and *P* < 0.05 indicated that the difference was statistically significant. The MCODE plugin in Cytoscape 3.4.0 is an APP that performs topological gathering on a fixed net to find dense connection areas. All differentially expressed genes enriched in five Kyoto Encyclopedia of Genes and Genomes (KEGG) pathways were introduced into the software. In addition, the most central modules in the PPI network were confirmed by MCODE.

### 2.5. Verify the Expression of Target Genes

Gene expression profile information of the 11 key genes in the sequence was imported into the cBioPortal website for genetic variation to verify the regulatory relationship between pivotal genes and miR-1-3p. In addition, the GraphPad Prism 8 software was used to verify the association between miR-1-3p and key genes.

## 3. Results

### 3.1. miR-1-3p Expression Decreased in HNSCC in miRNA-Seq

**Figure 2:**
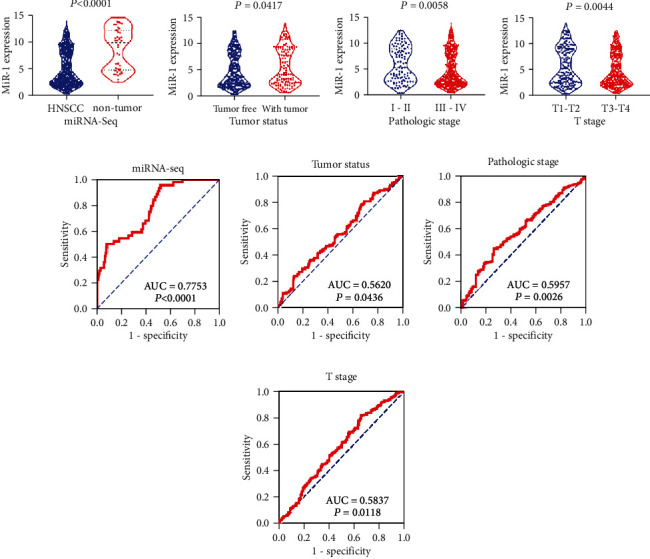
The miR-1-3p expression decreases in different clinicopathological parameters of HNSCC: (a–d) violin figure; (e–h) ROC curve. (a, e) The total expression of miR-1-3p in HNSCC and nontumor tissue from the TCGA database. (b, f) The relationship between miR-1-3p expression and tumor status. (c, g) The relationship between miR-1-3p expression and pathologic stage. (d, h) The relationship between miR-1-3p expression and T stage. The differential expression of miR-1-3p in HNSCC was statistically significant, which was manifested in tissue, tumor status, pathological stage, and T stage. AUC: the area under the ROC curve; *P* value: *T*-test with two independent samples.

In total, 44 nontumor and 484 HNSCC tissue samples were included in the miRNA sequence. Compared with noncancerous tissues, the expression of miR-1-3p in HNSCC visibly decreased (5.139 ± 3.275 vs. 8.709 ± 3.788, *P* < 0.001) (Figure 2(a)). The area under the ROC curve (AUC) was 0.775 (*P* < 0.001) (Figure 2(e)). From the statistical analysis of clinical parameters, the expression of miR-1-3p in tumor tissues showed distinct differences in tumor state (*P* = 0.0417, Figure 2(b)), pathological stage (*P* = 0.0058, Figure 2(c)), and T stage (*P* = 0.0044, Figure 2(d)) (Table 1).

### 3.2. Expression and ROC Curves of miR-1-3p in HNSCC in Different Gene Chips

**Figure 3:**
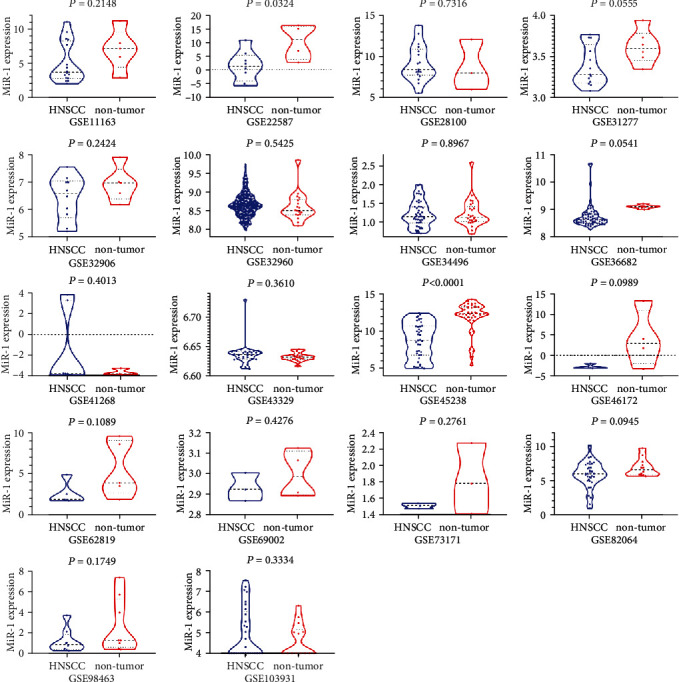
Expression of miR-1-3p in head and neck squamous cell carcinoma and noncancerous tissues in different gene chips.

**Figure 4:**
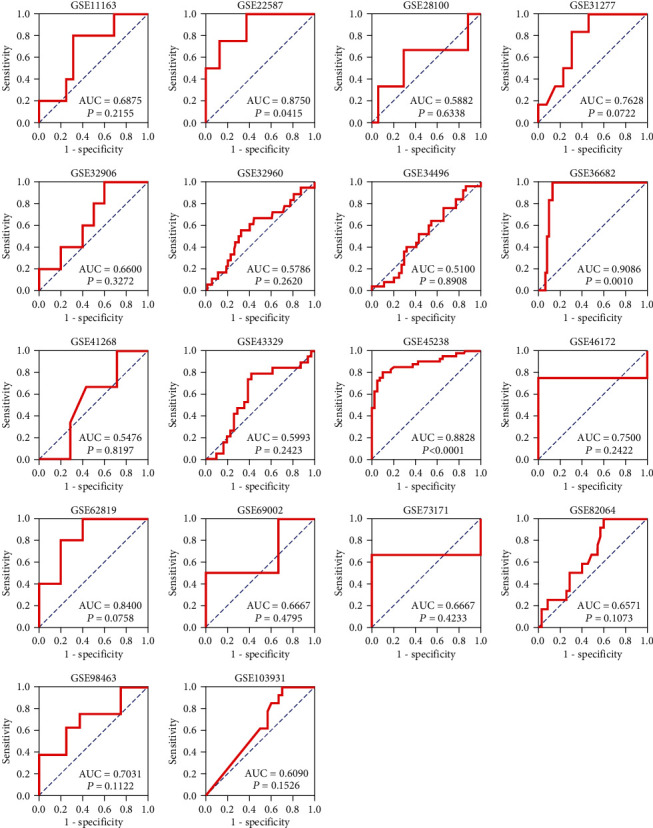
ROC curves of miR-1-3p in HNSCC and nontumor tissues in different gene chips.

Eighteen pieces of qualified chips were filtered from GEO, ArrayExpress, Oncomine, and SRA databases (Table 2). We calculated the mean and the SD in microarrays (Table 3) and plotted violin diagrams (Figure 3). We used adoptive data to calculate the AUC (Figure 4). Foregoing chip results revealed that the miR-1-3p expression level in HNSCC was lower than in noncancer cells, in accordance with the results from the sequence.

### 3.3. Meta-analysis of miR-1-3p Expression Levels in HNSCC Decreased Compared to Nontumor Tissues

**Figure 5:**
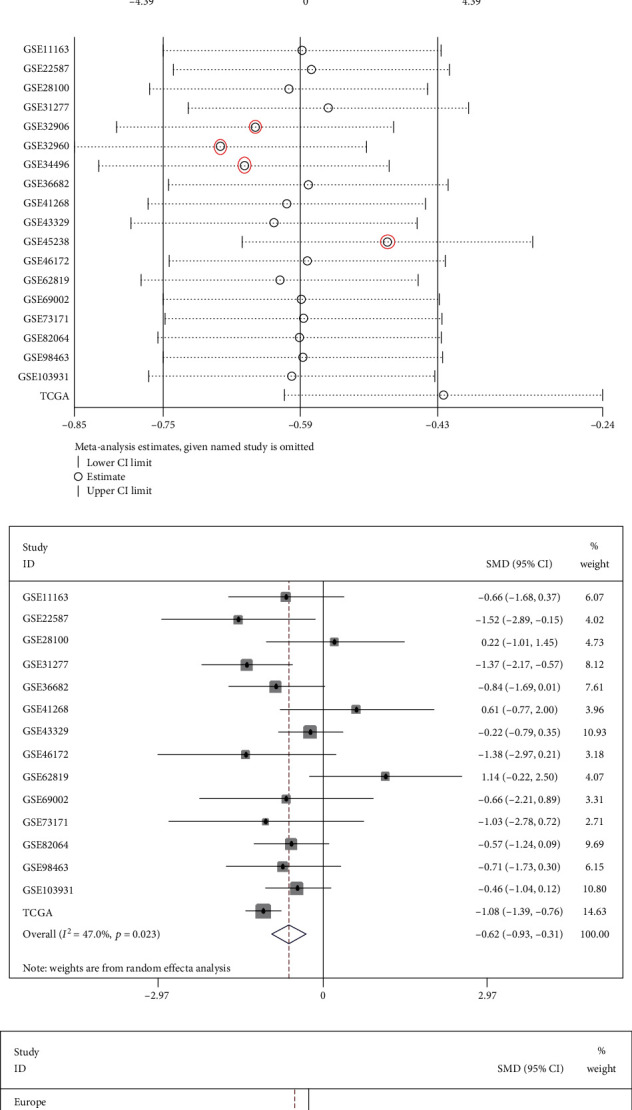
The meta-analysis of miR-1-3p expression levels in HNSCC decreases compared to nontumor tissues. (a) Forest map of SMD (fixed-effect model). (b) Forest plot of SMD (random-effect model). (c) The sensitivity analysis. (d) After the heterogeneity studies were eliminated, the forest plot of SMD based on 15 microarrays. (e) Subgroup analysis of countries was carried out to further explore the sources of heterogeneity. (f) Begg's funnel plot showed no obvious publication bias.

Detailed and comprehensive statistical analyses of data from the sequence and chips were performed to calculate the SMD values and to draw the forest plot (SMD = −0.59 (-0.75, -0.43), *I*^2^ = 78.5%, *P* < 0.001, Figure 5(a)). Data heterogeneity was overt. The random-effect model was used (Figure 5(b)) (SMD = −0.41 (-0.80, -0.03), *I*^2^ = 78.5%, *P* < 0.001). In the figure, the diamond occupied left of the invalid vertical line, miR-1-3p was weakly expressed in HNSCC, and the difference was statistically significant. Further sensitivity analysis was performed (Figure 5(c)). We selected four most influential studies to detect possible sources of heterogeneity (Figure 5(d)). Results showed that *I*^2^ = 47.0%, *P* = 0.023, heterogeneity decreased obviously, and the difference was statistically significant. This heterogeneity might come from these four chips: GSE32906, GSE32960, GSE34496, and GSE45238. The European SMD value was -0.28 (95% CI: -0.60, 0.04) (*I*^2^ = 0.0%), and the Asian SMD value was -0.32 (95% CI: -0.92, 0.28) (*I*^2^ = 83.7%) (Figure 5(e)), suggesting that heterogeneity may be derived from the country subgroup. The result of Begg's test was *P* = 0.961, indicating that there was no apparent publication bias in our study (Figure 5(f)).

### 3.4. Clinical Significance and Value of miR-1-3p in HNSCC

**Figure 6:**
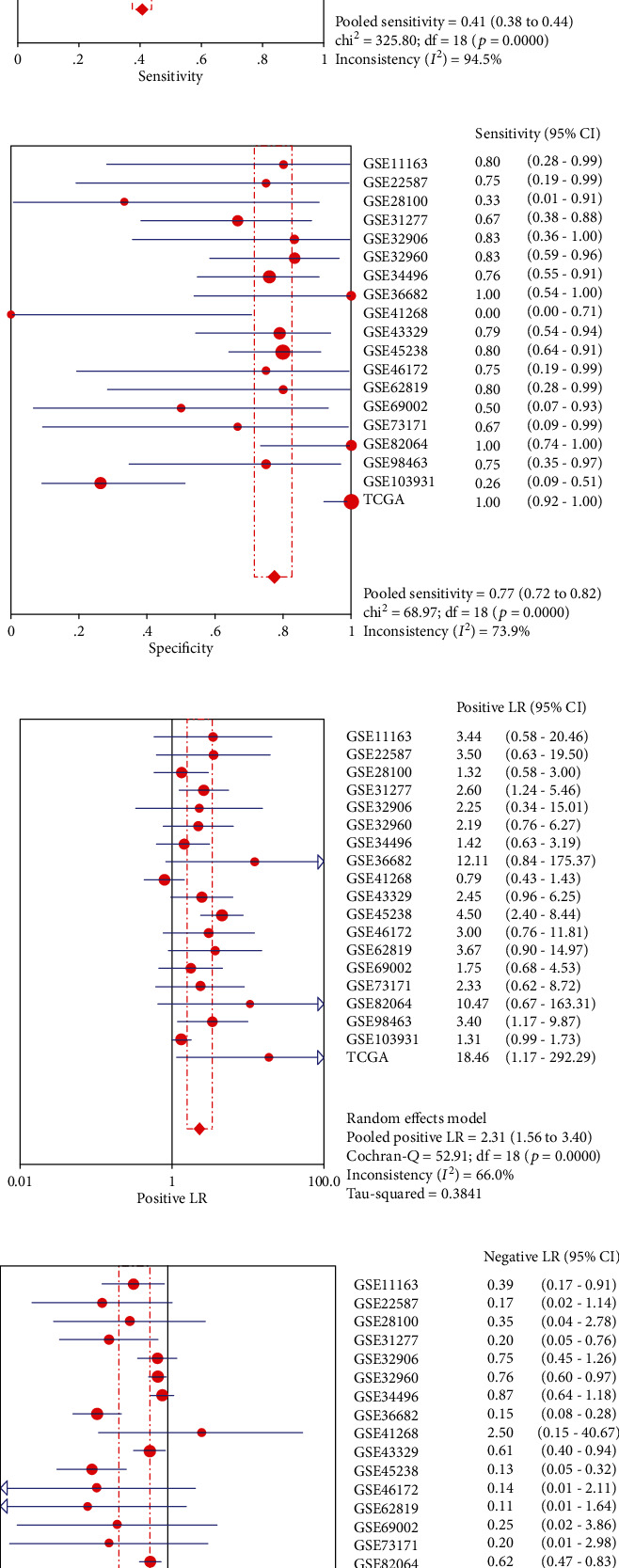
The values of total sensitivity, total specificity, positive likelihood ratio (PLR), negative likelihood ratio (NLR), diagnosis rate (DOR), and 95% confidence interval are statistically significant. (a) The SROC curve of miR-1-3p expression based on 19 datasets. (b–f) The forest map showed the diagnostic performance of miR-1-3p in HNSCC: the sensitivity of the collection, the specificity of the collection, the positive likelihood ratio of the summary, the negative likelihood ratio of the summary, and the summary diagnostic ratio based on the qualified dataset.

We computed the true positive (TP), false positive (FP), false negative (FN), and true negative (TN) of each dataset based on the most approximate den index and the corresponding cutoff value (Table 3). The area under the sROC curve was 0.83 (95% CI: 0.80-0.86) (Figure 6(a)). The values of total sensitivity, total specificity, positive likelihood ratio (PLR), negative likelihood ratio (NLR), diagnosis rate (DOR), and 95% confidence interval were 0.41 (95% CI: 0.38-0.44) (Figure 6(b)), 0.77 (95% CI: 0.72-0.82) (Figure 6(c)), 2.31 (95% CI: 1.56-3.40) (Figure 6(d)), 0.40 (95% CI: 0.26-0.61) (Figure 6(e)), and 7.87 (95% CI: 4.22-14.69) (Figure 6(f)), respectively.

### 3.5. Gene Enrichment Analysis of miR-1-3p in HNSCC

**Figure 7:**
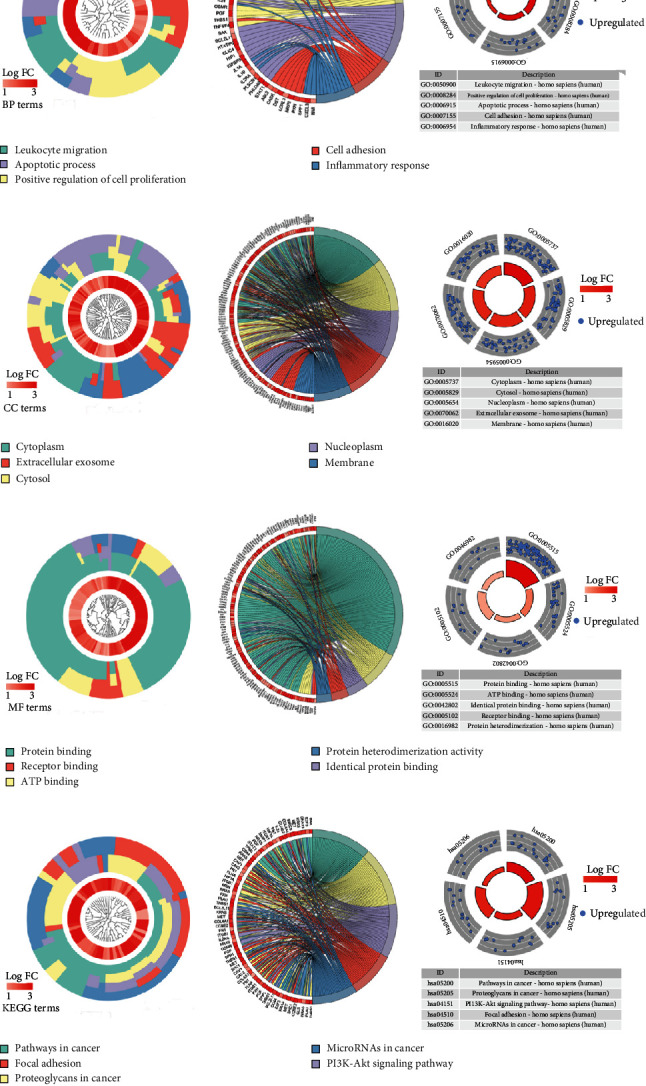
Gene enrichment circles of miR-1-3p in HNSCC: (a) biological process; (b) cellular component; (c) molecular function; (d) KEGG pathways.

**Figure 8:**
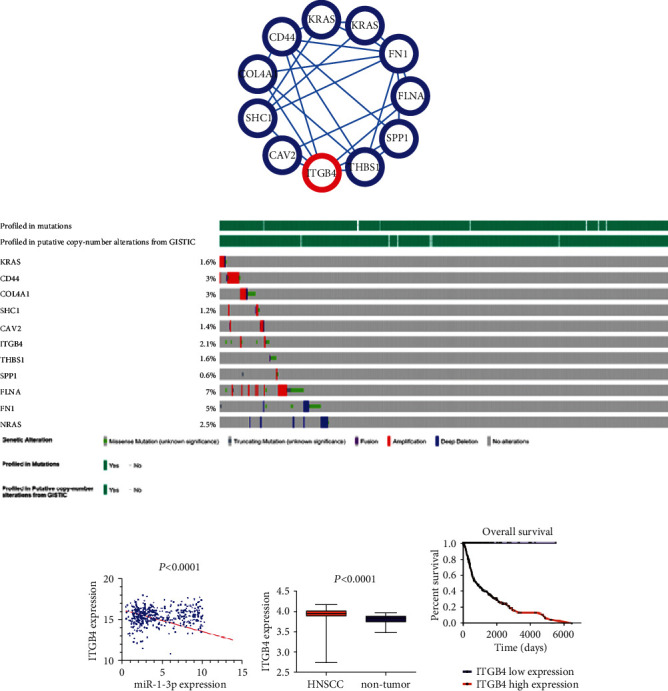
Preliminary prediction of ITGB4 as target gene of miR-1-3p. (a) Protein interaction network of 11 hub proteins. (b) 11 proteins were genetically altered in the HNSCC based on cBioPortal website. (c) Pearson correlation analysis showed that miR-1-3p was negatively correlated with ITGB4 (*P* < 0.0001). (d) ITGB4 expressed highly in HNSCC tumor tissues than in noncancer tissues. (e) Kaplan-Meier survival curve was used to analyze the ITGB4 expression data and evaluate its effects on the prognosis of HNSCC. ITGB4 had an apparent influence on the survival of HNSCC patients (*P* < 0.0001).

According to the logFC and *P* value, 174 upregulated differentially expressed genes and 103 downregulated genes were screened. Given that miR-1-3p was lowly expressed in HNSCC, we chose 174 upregulated DEGs for GO annotation and KEGG enrichment by DAVID 6.8 (*P* < 0.05), whose top five enriched terms were summarized according to *P* value (Table 4). Leukocyte migration, positive regulation of cell proliferation, apoptotic process, cell adhesion, and inflammatory response were the five most conspicuous terms for biological process (BP). In the cellular component (CC), genes were major enriched in the cytoplasm, cytosol, nucleoplasm, extracellular exosome, and membrane. As for molecular function (MF), the coexpressed proteins were involved in protein binding, ATP binding, identical protein binding, receptor binding, and protein heterodimerization activity. For the KEGG pathway, the coexpressed genes were major gathered in pathways in cancer, proteoglycans in cancer, PI3K-Akt signaling pathway, focal adhesion, and microRNAs in cancer (Figure 7). In these genes, gene networks showed that *KRAS*, *CD44*, *COL4A1*, *SHC1*, *CAV2*, *ITGB4*, *THBS1*, *SPP1*, *FLNA*, *FN1*, and *NRAS* were closely connected in the pathways (Figure 8(a)).

### 3.6. Preliminary Prediction of ITGB4 as Target Gene of miR-1-3p

The results of cBioPortal showed that the 11 target genes had different degrees of variation in HNSCC, reflected in a missense mutation, gene fusion, gene amplification, and gene deletion (Figure 8(b)). Only 2 HNSCC articles mentioned *ITGB4* and showed that it may be the target of HNSCC, which can promote distant metastasis of tumors through the blood. Pearson correlation analysis showed that miR-1-3p had a correlation with *ITGB4*, which was statistically significant (*P* < 0.001) (Figure 8(c)). For this reason, this gene was selected for further verification, and special attention was focused on the expression and prognosis of ITGB4 in HNSCC (Figures 8(d) and 8(e)).

## 4. Discussion

The downregulation of miR-1-3p in HNSCC was supported by 1,140 HNSCC and 243 noncancer tissue samples from TCGA, GEO, Oncomine, ArrayExpress, and SRA databases, which enhanced the dependability of our results. miR-1-3p's low expression could be associated with the burgeoning of HNSCC. To study the functional implication of miRNA-1 in HNSCC cells and identify new neoplastic paths, a more reliable set of target genes was obtained by integrating potential target genes composed of four parts: miR-1-transfected DEGs, sequence DEGs, chip DEGs, and the targets in the prediction tool. KEGG pathway analysis showed the most significant pathways were “the pathways in cancer,” “proteoglycans in cancer,” “PI3K-Akt signaling pathway,” “focal adhesion,” and “MicroRNAs in cancer.” We confirmed pivotal targets of miR-1-3p. *ITGB4* was one of the most important targets.

In recent years, research on noncoding RNAs appears to have advanced rapidly, especially the inquiry of miRNAs. A large number of miRNAs exert their functions in human diseases [26–28]. Most miRNAs serve as tumor suppressors for human cancers, such as miR-874 [29, 30], miR-21[31, 32], and miR-155 [33], while some miRNAs are overexpressed in human cancer tissues, such as miR-93, miR-218, and miR-375. Likewise, many miRNAs regulate miRNA expression and promote or suppress HNSCC cell proliferation [1, 34]. miR-1-3p was proven dysregulated in HNSCC [16, 35]. Nohata et al. [15] demonstrated that miR-1 was downregulated in HNSCC samples and disclosed that transgelin 2 (*TAGLN2*) was directly adjusted by miR-1. Koshizuka et al. [1] verified gene expression in HNSCC and found that miR-1 was reduced clearly in HNSCC tissues. However, only eight studies have investigated miR-1 expression in HNSCC. Further studies are needed to determine the relationship between miR-1 and HNSCC.

The 174 upregulated differentially expressed genes were screened from 39 datasets, three miR-1 transfection samples, and a prediction tool using the R language tool. Although the gene list was not large, screening was rigorous. The obtained difference genes were accurate and had a certain degree of persuasion. The KEGG enrichment pathway is involved in multifarious cancer processes. G protein-coupled receptors (GPCRs) play a vital role in signal transmission [36, 37]. Targeted proteins recognize and bind to binding sites in eukaryotes and activate a series of signaling pathways, causing changes in tumor cell states, promoting tumor blood vessel regeneration, and participating in the occurrence and course of neoplasms [38–41]. Therefore, understanding the specific mechanism of GPCR involvement in malignant tumors and related target genes is conducive to providing new opportunities for cancer prevention and treatment [36, 37]. Some studies have explored this pathway in bladder cancer [42, 43], colorectal cancer [38], melanoma [44, 45], endometrial cancer [46], lung cancer [47], renal cell carcinoma [48, 49], and thyroid cancer [50, 51]. Moreover, Koshizuka et al. found that miR-1 inhibited tumor growth by targeting growth factor receptors and participated in various signaling pathways, including the “pathways in cancer,” which was consistent with our results [1].

As key genes corresponding to important action sites, which contained important signal pathway information, we focused on hub genes in further research. Eleven hub genes were enriched in KEGG pathways, most of which had been reported by a large number of previous studies on HNSCC. ITGB4 caught our attention. The results showed that *ITGB4* was highly expressed in HNSCC and was harmful to patient prognosis (*P* < 0.001).

Integrin family, a family of cell adhesion receptors, is recognized to play a key role in malignant tumor metastasis [52]. As a component of the basement membrane, the expression levels of laminin-5 and its ligand were negatively correlated with tumor invasiveness, metastasis, and poor clinical prognosis [53–55]. ITGB4 encodes a receptor for laminin-5. Studies have shown that the decreased expression of ITGB4 and laminin-5 genes occurs during the progression of prostate intraepithelial neoplasia and the development of prostate cancer [56]. Meanwhile, *ITGB4* can be used as a target site to form a lump in colorectal cancer [57], gastric cancer [58, 59], prostate cancer [60–62], lung cancer [63], and other diseases [64, 65] to regulate the progress of diseases. A single study explored the extracellular matrix- (ECM-) receptor interaction, and *ITGB4* can be an underlying target for the diagnosis and treatment of HNSCC [66]. Through PCR analysis of oral squamous cell carcinoma data and assessment of pathological clinical parameters, Nagata et al. [67] found that ITGB4 could promote distant metastasis of tumors. ITGB4 is a good biological indicator of tumors. In this study, we used bioinformatics methods to speculate that ITGB4 gene can influence the disease course of HNSCC, and ITGB4 gene was correlated with miR-1-3p. In the treatment of HNSCC, this feature could be used to develop an inhibitor of ITGB4 for treatment of HNSCC.

Our research aimed to integrate microarrays and miRNA sequencing to study the expression and deep-seated mechanism of miR-1-3p in HNSCC. There are limitations in this research. First, data came from online databases; thus, larger clinical samples are needed for further experimental inquiry. Second, the targeting relationship between miR-1-3p and *ITGB4* was preliminarily verified, but further experiments are needed before clinical application.

## 5. Conclusion

Our research confirms the downregulation of miR-1-3p in HNSCC, revealing that miR-1-3p can act on target genes, activate signaling pathways, and participate in the development of HNSCC. ITGB4 may be a novel biological target protein, which requires further experimentation.

## Figures and Tables

**Table 1:** The relationship between miR-1-3p gene expression and clinical parameters in TCGA by using *T*-test.

Parameters	*N*	Mean ± SD	*T* value	*P* value
Tissue				
HNSCC	484	5.139 ± 3.274	6.048	0.0001
Normal	44	8.709 ± 3.788		
Age				
≥60	267	5.027 ± 3.259	0.756	0.4500
<60	214	5.253 ± 3.275		
Gender				
Male	350	5.099 ± 3.284	0.465	0.6420
Female	132	5.255 ± 3.267		
Lymphovascular invasion				
Yes	113	5.897 ± 3.548	1.702	0.0905
No	211	5.223 ± 3.314		
Tumor status				
With tumor	124	5.649 ± 3.467	1.967	0.0417
Tumor free	310	4.941 ± 3.177		
Histological grade				
G3-G4	122	5.307 ± 3.487	0.382	0.7022
G1-G2	340	5.175 ± 3.204		
Pathologic stage				
III-IV	376	4.924 ± 3.239	2.771	0.0058
I-II	106	5.915 ± 3.308		
T stage				
T3-T4	299	4.814 ± 3.069	2.762	0.0044
T1-T2	172	5.702 ± 3.515		
N stage				
N1-N3	239	5.055 ± 3.268	0.763	0.4465
N0	225	5.287 ± 3.274		
M stage				
M1	5	2.968 ± 1.602	3.043	0.0541
M0	457	5.198 ± 3.279		
Margin status				
Positive	55	5.246 ± 3.219	0.457	0.6480
Negative	322	5.467 ± 3.344		

**Table 2:** The gene chip dataset information of miR-1-3p.

Chip name	First author	Country	Public year	Sample	Platform
GSE11163	Michele Avissar	USA	2008	Tissue	GPL6690
GSE22587	Yang Shu	China	2013	Tissue	GPL8933
GSE28100	Hyunmin Jung	USA	2012	Tissue	GPL1085
GSE31277	Patricia Severino	Brazil	2014	Tissue	GPL4133
GSE32906	Zhaohui Luo	China	2012	Tissue	GPL11350
GSE32960	Jun Ma	China	2012	Tissue	GPL14722
GSE34496	Michael F Ochs	USA	2013	Tissue	GPL8786
GSE36682	Rongrong Wei	China	2012	Tissue	GPL15311
GSE41268	Zijun Xie	China	2012	Tissue	GPL10850
GSE43329	Jinze Zheng	China	2013	Tissue	GPL16475
GSE45238	Shine-Gwo Shiah	China	2015	Tissue	GPL8179
GSE46172	Jeffrey Bethony	USA	2014	Tissue	GPL16770
GSE62819	Jugao Fang	China	2014	Tissue	GPL16384
GSE69002	Chad Creighton	USA	2016	Tissue	GPL18044
GSE73171	Zenghong Li	China	2016	Tissue	GPL14613
GSE82064	Nicola Valeri	Switzerland	2017	Tissue	GPL21968
GSE98463	Cintia Micaela Chamorro	Spain	2017	Tissue	GPL21572
GSE10393	Yujin Hoshida	USA	2017	Tissue	GPL3921

**Table 3:** Basic statistical indicators of miR-1-3p expression values in the experimental groups and control groups were summarized.

Name	Case_n	Case_mean	Case_sd	Cont_n	Cont_mean	Cont_sd	TP	FP	FN	TN
GSE11163	16	5.0060	2.9502	5	6.9577	3.0365	11	1	5	4
GSE22587	8	1.3000	5.5401	4	10.1731	6.4789	7	1	1	3
GSE28100	17	9.1189	2.1971	3	8.6146	3.0807	15	2	2	1
GSE31277	15	3.4416	0.2865	15	3.7763	0.1914	13	5	2	10
GSE32906	16	7.7991	2.3468	6	1.2160	1.4288	6	1	10	5
GSE32960	312	8.6397	0.3116	18	8.5930	0.3889	211	8	101	10
GSE34496	44	1.2115	0.3470	25	1.2232	0.3832	15	6	29	19
GSE36682	62	8.7352	0.4357	6	9.0868	0.0713	54	0	8	6
GSE41268	7	-1.1088	3.6207	3	-3.0293	0.3337	2	0	5	3
GSE43329	31	6.5909	0.2343	19	6.6317	0.0071	17	4	14	15
GSE45238	40	8.6857	2.4182	40	11.9760	2.0615	36	8	4	32
GSE46172	4	-2.8090	0.5321	4	3.9720	6.9307	3	0	1	4
GSE62819	5	4.2859	3.0773	5	3.7886	3.0131	5	4	0	1
GSE69002	3	2.9315	0.0689	4	2.9969	0.1152	3	2	0	2
GSE73171	3	1.5082	0.0336	3	1.8233	0.4318	3	1	0	2
GSE82064	35	5.9177	1.9298	12	6.9437	1.2942	14	0	21	12
GSE98463	8	1.8867	1.9029	8	2.0199	2.4693	8	7	0	1
GSE103931	30	5.0757	1.3494	19	5.9368	2.4772	29	14	1	5

^a^Case_n, Case_mean, Case_sd: number, mean, standard deviation of experimental groups; Cont_n, Cont_mean, Cont_sd: number, mean, standard deviation of control groups; TP, FP, FN, TN: true positive, false positive, false negative, and true negative.

**Table 4:** Gene ontology analysis of DEGs involved in biological process, cellular component, molecular function, and KEGG pathways.

Category		Term	Genes	Count	*P* value
Biological process	GO:0050900	Leukocyte migration	CD44, KRAS, LYN, SHC1, CAV1, FN1, ITGB1, MIF, MMP1, MSN, MYH9, NRAS, OLR1, SLC7A11, SLC7A5, SLC7A8	16	4.40*E*-13
	GO:0008284	Positive regulation of cell proliferation	E2F3, FOSL1, KRAS, LYN, SHC1, TTK, ADM, BIRC5, CSNK2A1, FN1, ITGB1, IL24, OSMR, PGF, THBS1, TNFSF4	16	3.00*E*-05
	GO:0006915	Apoptotic process	BAX, BCL2L11, HTATIP2, BIRC5, CSNK2A1, CLIC4, HIP1, IGFBP3, IL1A, IL1B, IL2RA, IL24, PLSCR1, PHLDA2, STAT1, SULF1	16	2.60*E*-04
	GO:0007155	Cell adhesion	ABL2, CD44, CASK, DST, FN1, LOXL2, NRP2, OLR1, PXN, SPP1, THBS1, TROAP	12	3.80*E*-03
	GO:0006954	Inflammatory response	CXCL3, LYN, NMI, IL1A, IL1B, IL2RA, IL24, MIF, OLR1, SPP1, THBS1	11	2.90*E*-03

Cellular component	GO:0005737	Cytoplasm	BAX, CD44, CDC42BPA, CDC42EP3, POLD1, E2F3, ERCC6L, FRMD4A, FGD6, GNA13, GINS4, HAUS2, HTATIP2, KRAS, LYN, NMI, NUDCD1, OIP5, PDLIM7, PPFIA1, RAD54B, RECQL, TTK, WDHD1, ADM, BIRC5, B2M, CASK, CA2, CAPRIN1, CENPE, CLIC4, CCNA2, CDKN3, DTL, DNAH17, DST, EXO1, FTH1, FLNA, GJB3, GMPS, HIP1, HPRT1, HIF1A, ITGB1, MIF, MSN, MYO1B, MYO5A, MYH9, NASP, PXN, PSPH, PLAT, PHLDA2, PHLDB2, KCNS3, PCNA, PSMB9, RGS4, STAT1, SSH1, SLC7A5, SLC7A8, TACC3, TCOF1, TROAP	68	1.10*E*-04
	GO:0005829	Cytosol	ABL2, ATP6V1C1, BAX, BCL2L11, CDC42EP3, ERCC6L, ERF, FOSL1, HAUS2, KRAS, LYN, PPFIA1, SHC1, AP2M1, BIRC5, CASK, CA2, CSNK2A1, CAPRIN1, CDCA3, CENPE, CENPL, CENPN, CLIC4, CCNE2, DST, FTH1, FLNA, GLS, GMPS, HPRT1, HIF1A, IL1A, IL1B, MYO5A, MYH9, NDE1, PXN, PLSCR1, PSPH, PSMB9, RGS4, STAT1, SNRPF, SNRPG, SLC7A5, TGM2, TPM3	48	2.20*E*-04
	GO:0005654	Nucleoplasm	POLD1, DSCC1, E2F3, ERCC6L, ERF, GABPB1, GINS2, GINS4, HAUS2, HTATIP2, NMI, OIP5, POP1, RAD54B, RAD54L, RECQL, WDHD1, BIRC5, CSNK2A1, CENPL, CENPN, CCNA2, CCNE2, DTL, EXO1, HIF1A, KIF20A, LOXL2, MIF, NASP, NFYA, OLR1, PXN, PCNA, PSMB9, RFC3, STAT1, SSH1, SNAPC1, SNRPF, SNRPG, TBL1XR1, ZNF367	43	1.40*E*-04
	GO:0070062	Extracellular exosome	ATP6V1C1, BAX, CD276, CD44, CDC42BPA, GNA13, H2AFZ, LYN, AP2M1, AK2, B2M, CA2, CLIC4, DST, FTH1, FN1, FLNA, HPRT1, IGFBP3, ITGB1, IL1B, MIF, MSN, MYO1B, MYO5A, MYH9, MTMR11, NRAS, OLR1, PLSCR1, PLAT, PLAU, PCNA, PSMB9, SPP1, SERPINE1, SLC7A5, SLC7A8, SOD2, THBS1, TGM2, TMEM33, TPM3	43	1.80*E*-04
	GO:0016020	Membrane	ATP2B4, BAX, DDX18, POLD1, ERCC6L, GNA13, HTATIP2, KRAS, LARP4, RECQL, TTK, AGPS, ASPH, B2M, CAV1, CAV2, CAPRIN1, CENPE, CEP55, FLNA, HIP1, ITGB1, LOXL2, MYO5A, MYH9, NRAS, NRP2, NDE1, OLR1, PLSCR1, PGF, PHLDA2, SLC7A11, SLC7A5, SLC7A8	35	4.70*E*-04

Molecular function	GO:0005515	Protein binding	ABL2, ATP6V1C1, ATP2B4, BAX, BCL2L11, CD276, CD44, CDC42BPA, DDX18, POLD1, DSCC1, E2F3, ERCC6L, FOSL1, GNA13, GABPB1, GINS2, GINS4, H2AFZ, HTATIP2, KRAS, LYN, MET, NMI, NUDCD1, OIP5, PDLIM7, POP1, PPFIA1, RAD54B, RAD54L, RECQL, SHC1, TTK, WDHD1, YEATS2, AP2M1, ADM, AGPS, APOL1, ASPH, BIRC5, B2M, CASK, CA2, CSNK2A1, CAV1, CAV2, CDCA3, CENPE, CENPL, CEP55, CLIC4, COL4A1, CCNA2, CCNE2, CDKN3, DTL, DCBLD2, DST, ECE2, EXO1, FTH1, FN1, FLNA, GLS, HIP1, HPRT1, HIF1A, IGFBP3, ITGB1, IL1A, IL24, KIF20A, LOXL2, MIF, MAGOHB, MSN, MYH9, NASP, NRIP3, NFYA, NDE1, OLR1, PXN, PLSCR1, PGF, PLAT, PLAU, PHLDB2, PCNA, PSMB9, RFC3, SPP1, SCG5, SERPINE1, STAT1, SSH1, SNRPF, SNRPG, SLC7A11, SLC7A8, THBS1, TBL1XR1, TACC3, TGM2, TMEM33, TCOF1, TROAP, TPM3	110	5.30*E*-07
	GO:0005524	ATP binding	ABL2, ATP2B4, CDC42BPA, DDX18, ERCC6L, KRAS, LYN, MET, RAD54B, RAD54L, RECQL, TTK, AK2, CASK, CSNK2A1, CENPE, DYRK3, DNAH17, GMPS, KIF20A, MYO1B, MYO5A, MYH9, TGM2	24	7.70*E*-03
	GO:0042802	Identical protein binding	BAX, CDC42BPA, NMI, BIRC5, B2M, CAV1, FN1, HPRT1, NDE1, PCNA, STAT1, SOD2, THBS1	13	3.80*E*-02
	GO:0005102	Receptor binding	ABL2, CD276, LYN, ADM, CAV1, GRP, MIF, MSN, PLAT, SERPINE1, TNFSF4	11	1.40*E*-03
	GO:0046982	Protein heterodimerization activity	BAX, GABPB1, H2AFZ, BIRC5, CAV1, CAV2, HIP1, HIF1A, ITGB1, PGF	10	2.50*E*-02

KEGG PATHWAY	hsa05200	Pathways in cancer	BAX, E2F3, GNA13, KRAS, MET, BIRC5, COL4A1, CCNE2, IL24, FN1, HIF1A, ITGB4, MMP1, NRAS, PGF, STAT1, TPM3	17	5.90*E*-05
	hsa05205	Proteoglycans in cancer	CD44, KRAS, MET, CAV1, CAV2, FN1, FLNA, HIF1A, ITGB1, MSN, NRAS, PXN, PLAU, THBS1	14	6.30*E*-07
	hsa04151	PI3K-Akt signaling pathway	BCL2L11, KRAS, MET, COL4A1, CCNE2, FN1, ITGB1, IL2RA, NRAS, OSMR, PGF, SPP1, THBS1	13	8.10*E*-04
	hsa04510	Focal adhesion	MET, SHC1, CAV1, CAV2, COL4A1, FN1, FLNA, ITGB1, PXN, PGF, SPP1, THBS1	12	3.20*E*-05
	hsa05206	MicroRNAs in cancer	BCL2L11, CD44, E2F3, KRAS, MET, SHC1, CCNE2, GLS, NRAS, PLAU, THBS1	11	2.20*E*-03

^b^KEGG: Kyoto Encyclopedia of Genes and Genomes.

## Data Availability

The datasets generated and analyzed in the present study are available from the corresponding author upon reasonable request.
